# Genetic improvement of the shoot architecture and yield in soya bean plants via the manipulation of *GmmiR156b*

**DOI:** 10.1111/pbi.12946

**Published:** 2018-05-23

**Authors:** Zhengxi Sun, Chao Su, Jinxia Yun, Qiong Jiang, Lixiang Wang, Youning Wang, Dong Cao, Fang Zhao, Qingsong Zhao, Mengchen Zhang, Bin Zhou, Lei Zhang, Fanjiang Kong, Baohui Liu, Yiping Tong, Xia Li

**Affiliations:** ^1^ State Key Laboratory of Agricultural Microbiology College of Plant Science and Technology Huazhong Agricultural University Wuhan China; ^2^ State Key Laboratory of Plant Cell and Chromosome Engineering Institute of Genetic and Developmental Biology Chinese Academy of Sciences Beijing China; ^3^ University of Chinese Academy of Sciences Beijing China; ^4^ The Key Laboratory of Soybean Molecular Design Breeding Northeast Institute of Geography and Agroecology Chinese Academy of Sciences Harbin China; ^5^ Hebei Academy of Agriculture and Forestry Sciences Shijiazhuang China; ^6^ Anhui Academy of Agricultural Science Hefei China; ^7^ School of Life Sciences Guangzhou University Guangzhou China

**Keywords:** soya bean, GmmiR156b, SPL, shoot branching, plant architecture, yield

## Abstract

The optimization of plant architecture in order to breed high‐yielding soya bean cultivars is a goal of researchers. Tall plants bearing many long branches are desired, but only modest success in reaching these goals has been achieved. *MicroRNA156* (*miR156*)*‐SQUAMOSA PROMOTER BINDING PROTEIN‐LIKE* (*SPL*) gene modules play pivotal roles in controlling shoot architecture and other traits in crops like rice and wheat. However, the effects of *miR156*‐*SPL* modules on soya bean architecture and yield, and the molecular mechanisms underlying these effects, remain largely unknown. In this study, we achieved substantial improvements in soya bean architecture and yield by overexpressing *GmmiR156b*. Transgenic plants produced significantly increased numbers of long branches, nodes and pods, and they exhibited an increased 100‐seed weight, resulting in a 46%–63% increase in yield per plant. Intriguingly, *GmmiR156b* overexpression had no significant impact on plant height in a growth room or under field conditions; however, it increased stem thickness significantly. Our data indicate that *GmmiR156b* modulates these traits mainly via the direct cleavage of *SPL* transcripts. Moreover, we found that *GmSPL9d* is expressed in the shoot apical meristem and axillary meristems (AMs) of soya bean, and that *GmSPL9d* may regulate axillary bud formation and branching by physically interacting with the homeobox gene *WUSCHEL* (*WUS*), a central regulator of AM formation. Together, our results identify *GmmiR156b* as a promising target for the improvement of soya bean plant architecture and yields, and they reveal a new and conserved regulatory cascade involving *miR156*‐*SPL*‐*WUS* that will help researchers decipher the genetic basis of plant architecture.

## Introduction

Soya bean [*Glycine max* (L.) Merr.] is a major source of plant proteins and oils. As the demand for soya bean grows, the genetic improvement of soya bean cultivars to increase yields will become increasingly important (Teng *et al*., 2015). Shoot architecture is the most important trait of high‐yielding crops, and shoot branching is a major component of shoot architecture (Barbier *et al*., 2017; Mathan *et al*., 2016). Since the 1990s, a major goal of plant scientists has been to improve soya bean yields by breeding cultivars with optimal branching and an ideal shoot architecture. Three models are considered to define the ideal soya bean architecture (ISA): (i) tall plants with a large stature and at least five branches, (ii) intermediate plants with a moderate stature and two or three branches and (iii) compact plants with a small stature and one or two branches (Chavarria *et al*., 2017). Despite extensive effort, only modest improvement in soya bean architecture has been achieved. Further, no single gene has been identified to promote the ISA and high yields.

Soya bean has a unique plant architecture, with leaves, inflorescences and pods at each node. Therefore, the production of high‐yielding soya bean plants with an ISA requires coordination between branching (branch numbers, lengths and angles) and vertical growth (main stem‐containing nodes) (Pedersen and Lauer, 2004). Achieving the ISA has been an important research topic for decades, but the mechanism has remained elusive. Genomewide analyses using a homology‐finding approach have identified 406 genes that are potentially associated with branching in soya bean, and 57 of these genes are colocalized with quantitative trait loci for soya bean branching (Tan *et al*., 2013). However, no functional validation of the roles of these genes in soya bean architecture has been reported (Arite *et al*., 2007; Booker *et al*., 2004; Finlayson, 2007; Morris *et al*., 2001; Takeda *et al*., 2003; Zou *et al*., 2006).

Plant architecture is determined by axillary meristem (AM) activity and bud outgrowth (Wai and An, 2017; Wang and Jiao, 2018). As the activity of axillary buds is normally suppressed by the activity of the shoot apical meristem (SAM) (Domagalska and Leyser, 2011), plant architecture is controlled by the balance between the activities of the SAM and AM and is strictly regulated by genetic regulatory networks. To date, our knowledge of plant architecture comes mainly from studies of the model plants *Arabidopsis thaliana* (hereafter, Arabidopsis) and rice (*Oryza sativa*). In Arabidopsis, many genes involved in AM initiation (e.g. *LATERAL ORGAN BOUNDARIES1*) and lateral bud outgrowth (e.g. *BRANCHED1*) have been identified (Bell *et al*., 2012; Finlayson, 2007; Teichmann and Muhr, 2015). Notably, the homeodomain transcription factor WUSCHEL (WUS), which defines the shoot stem cell niche, is also involved in AM initiation (Wang *et al*., 2017). In rice, several genes, including *MONOCULM 1* and two *WUS* orthologs (*TILLERS ABSENT1* and *MONOCUM 3*), regulate tillering (Lu *et al*., 2015; Tanaka *et al*., 2015). Notably, the *SQUAMOSA PROMOTER BINDING PROTEIN‐LIKE* (*SPL*) family gene *OsSPL14*, also known as *IDEAL PLANT ARCHITECTURE1* (*IPA1*), is a key regulator of ideal rice architecture and high rice yields (Jiao *et al*., 2010; Miura *et al*., 2010; Zhang *et al*., 2017). *IPA1* is targeted by the microRNA (miRNA) *OsmiR156*, which is an upstream master regulator of ideal plant architecture (IPA) in rice.

MiRNAs are small noncoding RNAs (19–23 nt in length) that play crucial roles in plant development (Ha and Kim, 2014). *MiR156*s were originally identified as key regulators of the juvenile‐to‐adult phase transition in plants (Wang *et al*., 2009; Wu *et al*., 2009). Since then, their effects on plant architecture have been recognized in various species, including Arabidopsis and rice (Aung *et al*., 2014; Schwarz *et al*., 2008; Wang *et al*., 2015b). *MiR156*s affect shoot branching and plant architecture by cleaving *SPL* transcripts (Du *et al*., 2017; Liu *et al*., 2017; Wang *et al*., 2015b). Overexpression of *miR156*s promotes axillary bud initiation and outgrowth while suppressing SAM activity, causing a dwarf plant phenotype (Schwarz *et al*., 2008; Wang *et al*., 2015a). *MiR156* overexpression also modifies other traits, including flowering, root length and grain yields (Wang and Wang, 2015; Wang and Zhang, 2017; Yu *et al*., 2015). Thus, *miR156*‐*SPL* modules coordinately regulate yield‐related traits in crops. Soya bean has 28 *miR156*s in its genome; however, the roles of those *miR156*s in determining plant architecture and yield‐related traits are unknown.

Previously, we generated transgenic soya bean lines overexpressing *GmmiR156b* and confirmed the roles of *GmmiR156b* in flowering (Cao *et al*., 2015). Here, we report that *miR156b* is a master regulator of ISA and that overexpressing *GmmiR156b* promotes the first type of ISA, with a substantial yield increase per plant of up to 63%. *GmSPL9d* is the main target of *GmmiR156b*, and its encoded protein interacts directly with GmWUSs. We also found new high yield‐related roles for *GmmiR156b*, including in the development of soya bean leaves and stems and the determination of seed size. Our findings provide a promising avenue for the genetic improvement of soya bean architecture and novel insight into the molecular mechanisms underlying establishment of the ideal architecture and high yields in soya bean.

## Results

### *GmmiR156b* overexpression affects shoot branching but not plant height

**Figure 1:**
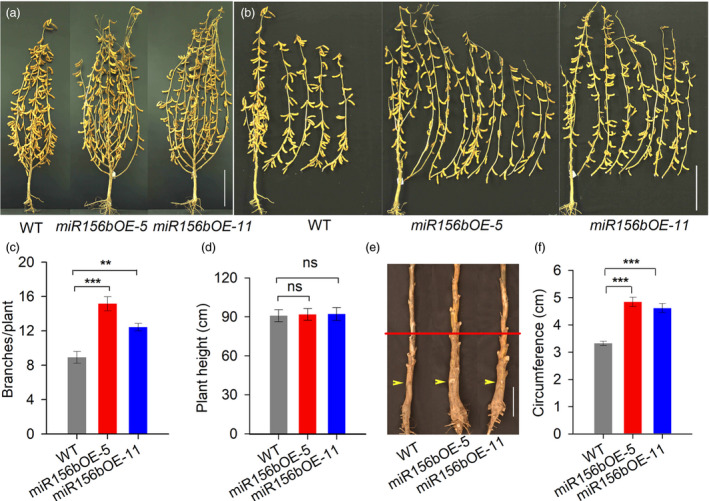
Characteristics of *miR156b* OE transgenic soya bean plants at the harvest stage. (a) Architecture of *miR156b* OE and wild‐type plants at the harvest stage. Scale bar, 20 cm. (b) Branching ability of the *miR156b* OE lines and wild type after harvest. Scale bar, 20 cm. (c) The mean total numbers of branches per *miR156b* OE plant and wild‐type plant (*n* = 20). (d) Heights of the *miR156b* OE lines and wild‐type controls (*n* = 20). (e) Stem morphology of *miR156b* OE and wild‐type plants at the harvest stage in the field. Yellow arrows show the sites of cotyledons at the postgermination stage. Red lines indicate the sites used to measure stem circumference in the transgenic and nontransgenic plants. Scale bar, 5 cm. (f) Statistical comparison of the stem circumference values of *miR156b* OE and wild‐type plants (*n* = 10). The data in the graphs represent means ± SEs. Statistical significance was estimated by Student's *t*‐tests. **P <* *0.01; ***P <* *0.001; ns, no significance.

To investigate whether *GmmiR156b* affects other traits in soya bean, we performed phenotypic studies using two transgenic lines with substantially increased levels of *GmmiR156b* (Figure S1) in a growth room and under natural field conditions (with a distance of 60 cm between rows and between the plants in each row). T_4_ transgenic plants of these *GmmiR156b*‐overexpressing (*miR156b*OE) lines showing varied levels of *GmmiR156b* expression exhibited a dramatically different shoot architecture compared with wild‐type controls. As shown in Figure 1, lines *miR156b*OE‐5 and *miR156b*OE‐11 grew bigger and had significantly more branches than nontransformed control plants at harvest time (Figure 1a,b). The average number of branches per transgenic plant was 12–15, while the average number of branches per control plant was only 8–9 (Figures 1c and S2). It is well known that planting density has a strong impact on branching and plant architecture. To assess whether the increased number of branches in the *miR156b*OE plants was due to the large interplant distance, we planted T_5_ plants in the same field with two different interplant distances (row and plant spacing: 40 cm or 50 cm). The *miR156b*OE plants were bigger and exhibited greater branching activity than wild‐type control plants regardless of the plant/row spacing (Figure S3). Our results suggest a pivotal role for *GmmiR156b* in the genetic control of branching in soya bean.

Interestingly, the heights of both transgenic lines were comparable to that of the wild‐type control plants, even though the lines showed different levels of *GmmiR156b* overexpression (Figure 1d). This is different from the dwarf phenotypes observed in other plants (Wang *et al*., 2015a). In addition, the *miR156b*OE transgenic plants had significantly sturdier stems than did the control plants (Figure 1e,f). Notably, the severity of the phenotypes of *miR156b*OE‐5 transgenic line was stronger than that of the *miR156b*OE‐11, indicating that the level of *GmmiR156b* expression may determine the phenotypes.

### *GmmiR156b* overexpression increased the yield per plant substantially

**Figure 2:**
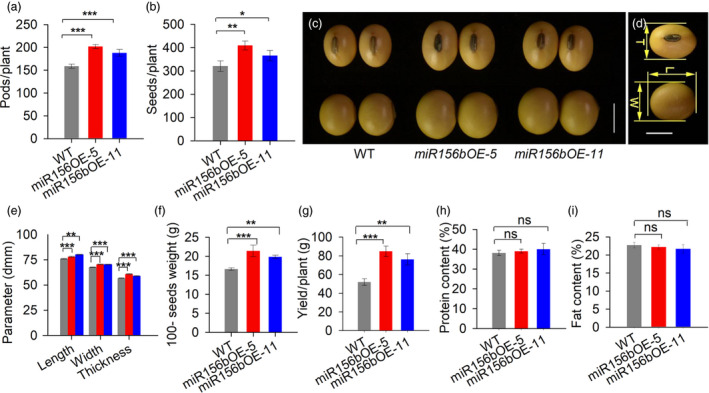
*GmmiR156b* overexpression can increase the grain yield per plant but does not alter seed quality. Total pod number (a) and seed number (b) per transgenic plant and wild‐type plant (*n* = 20) at the harvest stage. (c) Seed morphology of *miR156b* OE lines and wild type after harvest. Scale bar, 5 mm. (d) Soya bean seed characters and (e) size parameters of the *miR156b* OE lines and wild‐type control plants after harvest (*n* > 40). T, L and W are the abbreviations for seed thickness, length and width, respectively. Scale bar, 5 mm. (f) The 100‐seed weights of the *miR156b* OE lines and wild‐type control plants (*n* = 20). (g) Yield per plant in *miR156b* OE lines and wild type (*n* = 15). (h) The protein and lipid contents of seeds from *miR156b* OE and wild‐type plants. The data in the graphs represent means ± SEs. Statistical significance was estimated by Student's *t*‐tests. *P <* *0.05; **P <* *0.01; ***P <* *0.001; ns, no significance.

To determine the effects of *GmmiR156b* overexpression on yield, we characterized the pod number, seed number and 100‐seed weight per plant, which are major factors affecting soya bean yields. As shown in Figure 2, the total number of pods per transgenic plant was significantly higher than in the wild‐type control (Figure 2a). Accordingly, the average seed number per transgenic plant was increased by approximately 28% (Figure 2b). Furthermore, *GmmiR156b* overexpression had a large impact on seed size (including the length, width and thickness), resulting in bigger seeds compared with those of wild‐type control plants (Figure 2c–e) and a significant increase in the 100‐seed weight per plant (Figure 2f). Consequently, the highest seed yields per plant were obtained from the transgenic plants, and the average grain yield per plant among the *miR156b*OE plants was increased by 46% (*miR156b*OE‐11) to 63% (*miR156b*OE‐5) (Figure 2g). Intriguingly, the protein and lipid contents of the transgenic soya bean seeds were not significantly different compared with wild type (Figure 2h,i), suggesting a major role for *GmmiR156b* in determining soya bean yields.

### *GmmiR156b* overexpression shortens the plastochron of trifoliolate leaves

**Figure 3:**
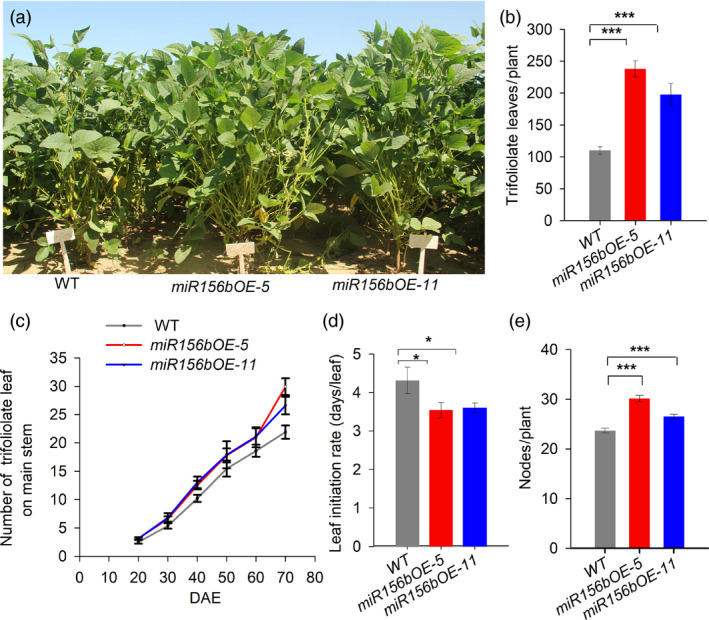
*GmmiR156b* overexpression shortens the length of the plastochron to increase the number of trifoliolate leaves and nodes per plant. (a) Transgenic *miR156b* OE‐5, *miR156b* OE‐11 and wild‐type control plants were grown in a field at 70 days after emergence (DAE). Scale bar, 20 cm. (b) The total trifoliolate leaf number per transgenic and wild‐type plant (*n* = 20). (c) Dynamic changes in the trifoliolate leaf number on the main stem in wild type and *miR156b* OE transgenic lines (*n* = 10). (d) The leaf initiation rates were significantly increased in the *miR156b* OE lines (*n* = 10). (e) Average node number per transgenic and wild‐type plant (*n* = 20). All data in the graphs represent means ± SEs. Statistical significance was estimated by Student's *t*‐tests. *P <* *0.05; ***P <* *0.001.

The plastochron was shorter in *miR156b*OE plants than in wild‐type plants. At 70 days after emergence (DAE), the number of trifoliolate leaves per *miR156b*OE plant was significantly higher than in wild type under field conditions (Figure 3a). The total number of trifoliolate leaves, including on the main stem and branches, per *miR156b*OE plant increased about 120%. In particular, the number of leaves on the branches was greatly increased compared with wild type because of the increased branch number (Figure 3b,c). Both *miR156b*OE lines grew faster than wild type in terms of trifoliolate leaf initiation (Figure 3d). Accordingly, the node number per *miR156b*OE plant was markedly increased (Figure 3e). Decreased plastochron lengths were also observed when the *miR156b*OE plants were grown indoors (Figure S4). The total number of trifoliolate leaves on the primary stem was significantly different between the *miR156b*OE and wild‐type control plants at 30 DAE (Figure S3). This observation is consistent with previous results obtained for Arabidopsis *miR156f* (Wang *et al*., 2008), suggesting that *GmmiR156* has a conserved function in leaf plastochron determination.

### *GmmiR156b* overexpression stimulates SAM and AM activity

**Figure 4:**
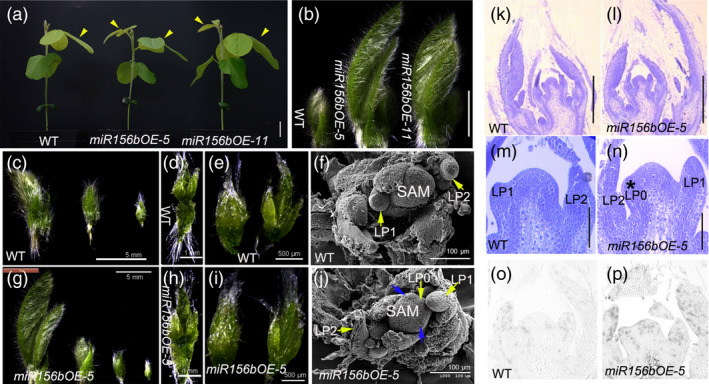
*GmmiR156b* overexpression enhanced the meristematic activity in transgenic plants at the vegetative growth stage. (a) Leaf phenotypes of *miR156b* OE lines grown in a growth room for 15 days after emerging from soil. Yellow arrows indicate trifoliolate leaves. Scale bar, 2 cm. (b) Shoot apical tissues from *miR156b* OE lines after removing two visible and mature leaves under a stereoscopic microscope. Scale bar, 5 mm. Dissected immature leaves from wild‐type (c–e) and *miR156b* OE‐5 (g–i) soya bean shoot apical tissues. (f and j) Scanning electron microscopic images of the shoot apical meristem (SAM) in wild‐type (f) and *miR156* OE‐5 (j) plants. Yellow arrows indicate leaf primordia (LP). The numbers represent LP at different developmental stages. Blue arrows show initiated stipule primordia. At least ten individuals were examined for each genotype; representative images are shown. (k–n) Morphological structure of a wild‐type (k and m) and *miR156b* OE‐5 SAM (l and n). Scale bars in k and l, 200 μm. Scale bars in m and n, 50 μm. (o and p) *In situ* hybridization analyses of *Histone H4* in wild‐type (o) and *miR156b* OE‐5 (p) shoot apices at 15 days after emergence.

To investigate the cytological basis for the regulatory effects of *GmmiR156* on the leaf plastochron and branching in soya bean, we performed the following experiments. First, we harvested shoot growth tips from potted *miR156b*OE and wild‐type plants (which exhibited a clear difference in trifoliolate leaf growth at 15 DAE; Figure 4a). As *miR156*OE‐5 line showed stronger shoot phenotypes, we used line *miR156b*OE‐5 for further morphological observation of the shoot tips [including leaf primordia (LP), the SAM and AMs]. As shown in Figure 4b, newly emerged trifoliolate leaves in the *miR156b*OE shoot apices were much bigger than those in wild type, and there was one more young trifoliolate leaf visible at the *miR156b*OE‐5 shoot apex than in wild type (Figure 4c,g). Although no significant differences were noted between the shoot apices of *miR156*OE‐5 and the control (Figure 4d,e,h,i), scanning electron micrographs showed different developmental characteristics in these shoot apices, including the number of LP and morphology of the SAM (Figure 4f,j). Longitudinal (paraffin) sections of the shoot apices confirmed that the overexpression of *GmmiR156b* promoted shoot development (Figure 4k,l). Notably, *GmmiR156b* overexpression also accelerated the initiation and growth of LP and axillary bud primordia (Figure 4m,n). There were three axillary bud primordia in the axil of the LP at the *miR156b*OE‐5 apex, compared to only two axillary bud primordia in the axil of the LP at the apex of the wild‐type plants. Meanwhile, LP were initiated in the SAM of the *miR156b*OE plants but not in the wild‐type control.

To determine whether *GmmiR156b* overexpression raised the meristematic activity of the SAM and AMs, we performed *in situ* hybridization for *Histone H4* mRNA to detect proliferating cells in S phase of the cell cycle (Gaudin *et al*., 2000). As expected, the level of *Histone H4* expression in the *miR156b*OE‐5 apex was dramatically higher than that in wild type (Figure 4o,p). These results demonstrate that *GmmiR156b* is a positive regulator of SAM and AM formation and development.

### *GmSPL9d* may play a major role in *GmmiR156b*‐mediated SAM and AM development

**Figure 5:**
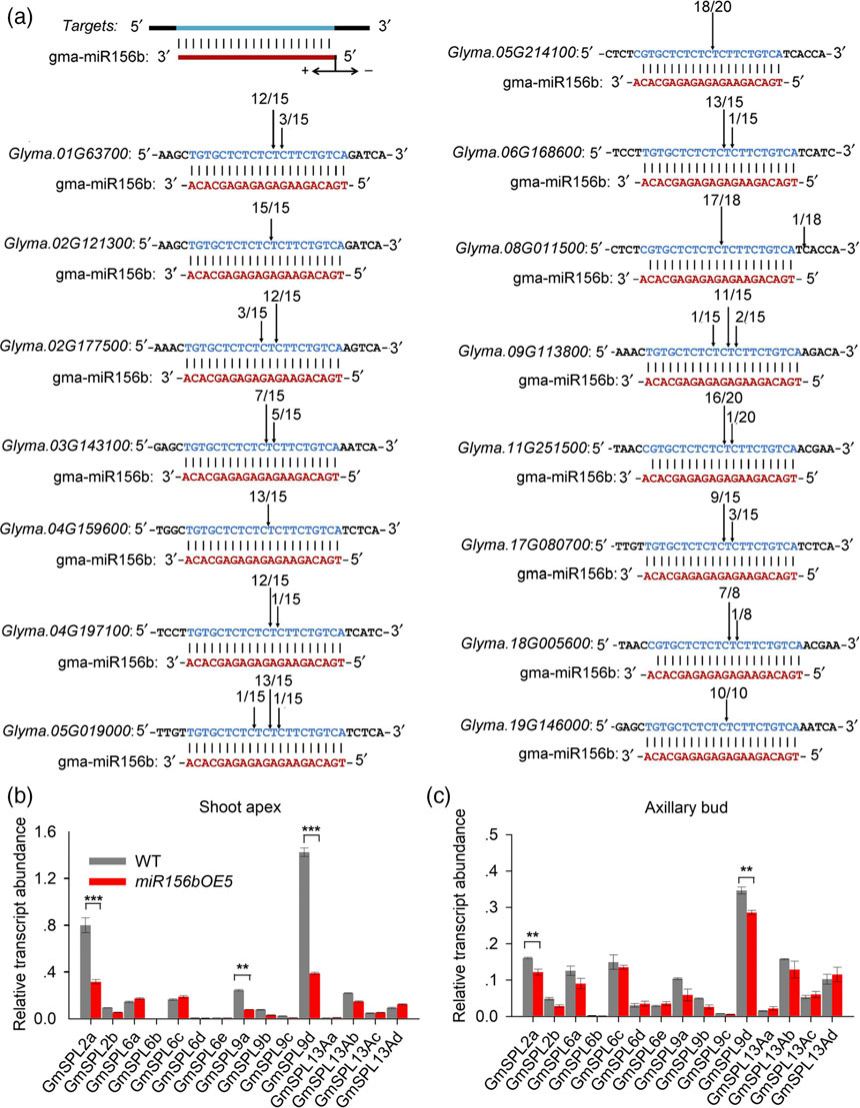
5′ RACE validation of the target genes of *GmmiR156b* and expression analyses. (a) Experimental validation of *miR156b* target genes and cleavage sites using 5′ RACE. Vertical arrows indicate the 5′ termini of the miRNA‐guided cleavage products, as identified by 5′ RACE, with the frequency of clones shown. (b and c) qRT‐PCR analysis of *GmSPL* expression in the shoot apex (a) and axillary buds (b) of *miR156b* OE‐5 and wild‐type plants. *GmELF1b* was used as an endogenous control for gene expression. Asterisks represent significantly decreased expression of the *GmSPL* genes in *miR156* OE‐5 plants (Student's *t*‐test; **P <* *0.01; ***P <* *0.001).

*MiR156*s mediate various biological processes through their *SPL* gene targets. In soya bean, 17 *SPL* genes, including two *GmSPL2*s, five *GmSPL6*s, four *GmSPL9*s and six *GmSPL13*s, were found (Table S1 and Figure S5). Among them, two *GmSPL13* genes (*GmSPL13Ba* and *GmSPL13Bb*) were predicted to be regulated by *GmmiR156b* at the translational level; the rest of the *GmSPL* mRNAs are likely cleaved by *GmmiR156b* because they contain sequences that are complementary to the miRNA (Figure 5a). Next, we performed a 5′ Rapid Amplification of cDNA Ends (RACE) assay and found that all of the 15 *GmSPL* mRNAs were efficiently cleaved between base pairs 10 and 11 of the *GmmiR156b* target sites (Figure 5a), suggesting that these *SPL*s are targeted by *GmmiR156b*.

To identify specific *SPL* genes targeted by *GmmiR156b* during SAM and AM development, we analysed the expression of 15 *GmSPL* genes in the shoot apex and axillary buds of *miR156b*OE‐5 and wild‐type plants at 15 DAE. Our qRT‐PCR results show that *GmSPL2a*,* GmSPL9a* and (especially) *GmSPL9d* were significantly down‐regulated in the shoot apex and axillary buds of *miR156b*OE‐5 plants, while the expression of other target genes was slightly down‐regulated or not affected (Figure 5b,c). This result indicates that *GmSPL2a*,* GmSPL9a* and *GmSPL9d* may be targets of *GmmiR156b* in the SAM and AM, and that *SPL9d* is likely the main target of *GmmiR156b*. Meanwhile, we also analysed the expression of 15 predicted targets in other organs and found that expression of these target genes showed different patterns of organ specificity. Among them, *GmSPL2a* was the most abundant target gene in flower; *GmSPL6a* was most abundant in stem, leaf and root, while *GmSPL6d* was most abundance in seed. Interestingly, *GmSPL9d* also the highest level of expression in nodule (Figures S6). Together, these results indicate that miR156b may regulate the IPA and high yield trait through different target genes in soya bean.

### *GmSPL9d* overexpression suppresses branching in Arabidopsis

**Figure 6:**
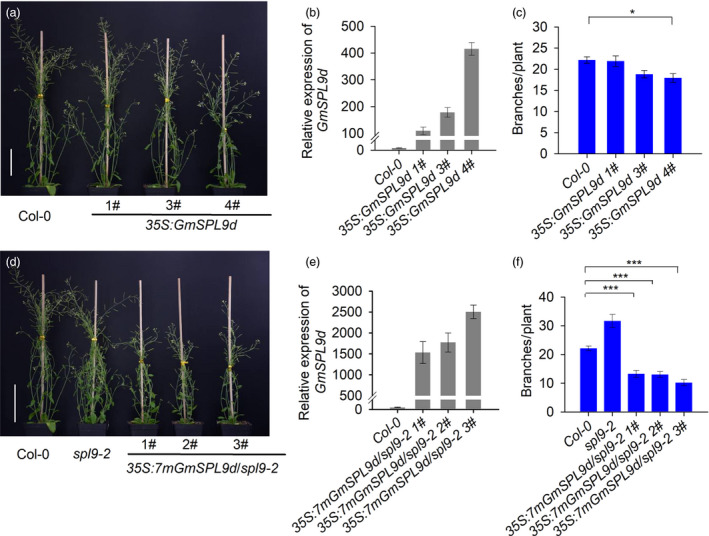
*GmSPL9d* has a conserved function in branching control. (a) Overexpression of *GmSPL9d* in Col‐0. Scale bar, 5 cm. (b) qRT‐PCR analysis of *GmSPL9d* expression in three transgenic Arabidopsis lines. (c) Branch number in Col‐0 and transgenic Arabidopsis lines overexpressing *SPL9d*. (d) Overexpression of the mutated gene (*7mGmSPL9d*) inhibited branching and the height of the *spl9‐2* mutant. Scale bar, 5 cm. (e) qRT‐PCR analysis of *GmSPL9d* expression in *spl9‐2* mutant plants ectopically expressing *GmSPL9d*. (f) Quantitative analysis of branch number in wild‐type plants (Col‐0) and *spl9‐2* mutant plants overexpressing *7mGmSPL9d* (*n* > 10). The data were surveyed at 30 days after emergence from soil. *ACTIN8* was used as an internal control for gene expression. All data are represented as means ± SEs. Asterisks represent a significantly decreased branch number in *spl9‐2* mutants overexpressing *7mGmSPL9d* compared with that in Col‐0 (Student's *t*‐test; *P <* *0.05; ***P <* *0.001).

To further test whether *GmSPL9d* functions in SAM and AM development, we ectopically expressed *GmSPL9d* under the control of the *35S* promoter in Arabidopsis ecotype Columbia 0 (Col‐0) plants. Only high levels of *GmSPL9d* expression significantly reduced the branching and height of Col‐0 plants (Figure 6a–c). This is likely due to the fact that *GmSPL9d* mRNAs are cleaved by Arabidopsis *miR156*s because *GmSPL9d* is phylogenetically similar to *AtSPL9*, and both genes possess the same *miR156b* target sequence (Figure S7).

Previously, it was shown that a loss of function in *AtSPL9* resulted in a dramatically increased number of branches and a bushy phenotype in Arabidopsis (Schwarz *et al*., 2008). To confirm the effect of *GmSPL9d* on shoot architecture, we overexpressed a mutated version of *GmSPL9d* (*7mGmSPL9d*) with seven‐point mutations at the *miR156b* cleavage site (mismatches) without affecting any amino acids in the *spl9‐2* mutant. As shown in Figure 6d–f, *GmSPL9d* was highly expressed in *spl9‐2‐*expressing *7mGmSPL9d* plants, and the *7mGmSPL9d*‐OE *spl9‐2* plants were significantly shorter than *spl9‐2* plants; moreover, the number of branches was significantly lower than in *spl9‐2* mutant and Col‐0 plants. Taken together, these results demonstrate that *GmSPL9d* has a conserved function in shoot branching in plants, but that it may have a distinct role in apical dominance in soya bean. Our data also indicate that the expression level of *GmSPL9d* is closely related to the activity of apical growth and branching.

### GmSPL9d interacts with GmWUSa/b

**Figure 7:**
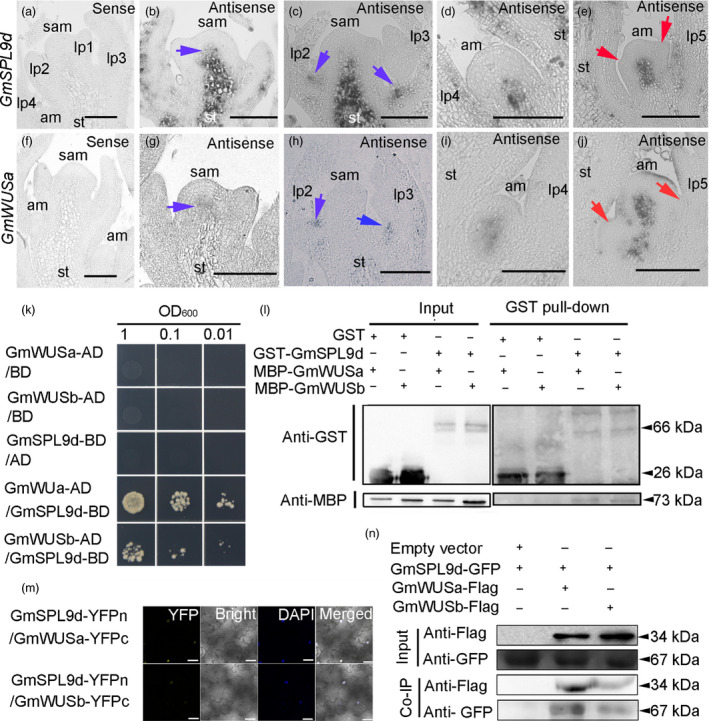
GmSPL9d interacts with GmWUS. (a–e) *In situ* expression patterns of *GmSPL9d* in the shoot apical meristem (SAM) and axillary meristems (AMs). (a) Vegetative shoot apices at 15 days after emergence (DAE) were hybridized with a *GmSPL9d* sense strand control. (b) The *GmSPL9d* antisense probe signal appeared in the SAM (blue arrow). (c) The *GmSPL9d* signal appeared in the leaf axillary region (blue arrows) at the pre‐AM formation stage. (d) The *GmSPL9d* signal was detected in AM bumps. (e) *GmSPL9d* was expressed in established axillary buds. Red arrows show leaf primordia (LP). (f–j) *In situ* expression of *GmWUSa* in the SAM and at different stages of axillary bud formation in wild type. (f) The vegetative shoot apex of plants at 15 DAE was hybridized with a *GmWUSa* sense strand control. (g) The *GmWUSa* antisense signal appeared in the SAM (blue arrow). (h) A *GmWUSa* signal also appeared in the leaf axillary region (blue arrows) at the pre‐AM formation stage. (i) The *GmWUSa* signal detected in the AM bumps where *GmSPL9d* was expressed. (j) *GmWUSa* expression in an established axillary bud. Red arrows indicate LP. *Note*: lp1–5 represent LP at different developmental stages. st, stem. Scale bar, 50 μm. (k) Y2H assay validating the interaction of GmSPL9d with GmWUSa/b. (l) A GST pull‐down assay showing the interaction between GmSPL9d and GmWUSa/b. The sizes of GST tag, GST‐GmSPL9d fusion protein, MBP tag and MBP‐GmWUSa/b fusion proteins were indicated. (m) BiFC in tobacco leaves. Visible light indicates the interaction between GmSPL9d and GmWUSa/b in the nucleus. DAPI staining was used as a nuclear marker. Panels (left to right): YFP; bright; DAPI staining; merged channels. Scale bar, 25 μm. (n) Co‐IP of GmSPL9d‐GFP and GmWUSa/b‐flag. Transgenic plants expressing GFP were used as a negative control. The sizes of Flag tag, GmWUSa/b‐Flag fusion protein, GFP tag and GmSPL9d‐GFP fusion protein were indicated.

We next explored the molecular mechanism by which GmSPLs affect soya bean plant architecture. Because WUSs are key regulators of both the SAM and AM in plants and GmWUSa is expressed in the SAM and AM and affects Arabidopsis shoot architecture (Tanaka *et al*., 2015; Xin *et al*., 2017), we speculated that two transcription factors (GmSPL9d and GmWUSa) work together to regulate soya bean shoot architecture. To test this, we first performed *in situ* hybridization. Our results show that *GmSPL9d* was coexpressed with *GmWUSa* in the SAM and AMs (Figure 7a–j). To further address the relationship between GmSPL9d and GmWUSa/b, we performed several protein interaction assays. Yeast two‐hybrid (Y2H) assays showed that GmSPL9d can physically interact with GmWUSa/b, but that the interaction with GmWUSa is stronger (Figure 7k). The physical interaction between GmSPL9d and GmWUSa/b was further confirmed by a pull‐down assay (Figure 7l). The interaction of GmSPL9d with GmWUSa/b was then confirmed using a bimolecular fluorescence complementation (BiFC) system (Figure 7m). To validate the GmSPL9d‐GmWUSa/b interaction, we performed co‐immunoprecipitation (Co‐IP) experiments using a transient expression system in *Nicotiana benthamiana* leaves. Constructs expressing GmSPL9d‐GFP and GmWUSa/b‐flag fusion proteins were cotransformed into tobacco. As shown in Figure 7n, the GmSPL9d‐GFP fusion protein was detected in cell extracts when it was coexpressed with GmWUS‐flag; by contrast, no protein interaction was detected when GmSPL9d‐GFP was coexpressed with myc‐flag. Our *in vitro* and *in vivo* results demonstrate that GmSPL9d can directly interact with GmWUSa/b.

To examine which domain of GmWUS is involved in its interaction with GmSPL9d, we fused the full‐length or truncated GmWUSa coding sequence to the Gal4‐DNA‐binding domain to produce bait vectors and fused the full‐length GmSPL9d coding sequence to the GAL4 activation domain (AD; GAL4‐AD‐GmSPL9d) to produce the prey vector. Y2H assays revealed that the acidic domain (amino acids 220–234) of GmWUSa is essential for the interaction between GmWUSa and GmSPL9d (Figure S8a), and the results were confirmed by BiFC assays (Figure S8b).

As SPL9 and WUS are markers of plant growth and architecture (Mayer *et al*., 1998; Wang *et al*., 2009), we questioned whether the SPL9‐WUS protein interaction is a conserved regulatory mode during plant growth and development. In Y2H and BiFC assays, SPL9 interacted strongly with WUS in Arabidopsis (Figure S9). To determine whether other SPLs interact with WUS, we included SPL2 in our assays. The results confirmed an interaction between SPL2 and WUS (Figure S10), indicating that SPL‐WUS interactions might be a conserved mechanism of SAM and AM regulation in plants.

## Discussion

The roles of *miR156* in branching have been shown in monocots (e.g. rice and wheat) and dicots (e.g. Arabidopsis and alfalfa). Overexpression of *miR156* increases branching, resulting in bushy plants (Chuck *et al*., 2007; Fu *et al*., 2012; Liu *et al*., 2017; Luo *et al*., 2012; Schwab *et al*., 2005; Xie *et al*., 2012). In monocots, tiller number is related to the number of spikes or panicles per plant. The fact that *miR156* overexpression causes an increase in tiller number and reduction in spike number (Liu *et al*., 2017; Wang *et al*., 2015a,b) has hindered the application of *miR156* in IPA breeding. In soya bean, branch number is directly correlated with the pod number per plant, and long branches with effective nodes are a key requirement for the first ISA model. Our finding that *GmmiR156b* overexpression dramatically increased the number of branches in soya bean (Figure 1a–c) supports the notion that *GmmiR156* is a central regulator of shoot branching in higher plants. Intriguingly, overexpression of *GmmiR156b* enabled soya bean plants to produce an increased number of long branches with effective nodes (Figure 1b), thereby resulting in increased numbers of pods and seeds, and a high yield per plant (Figure 2a,b). Previously, we reported that overexpression of *GmmiR156b* delayed flowering of soya bean for about 20 days in the controlled growth chamber (Cao *et al*., 2015). Such a long time delay in flowering will greatly affect maturation of plants and eventual yield in soya bean. Unexpectedly, these *GmmiR156b*OE plants only showed slight delay in flowering time under natural field conditions. The flowering time difference between the *GmmiR156b*OE plants grown in the controlled and natural conditions may be caused by the environmental factors, such as light, diurnal temperature variation and so on. Thus, *GmmiR156b* is an ideal target for the genetic improvement of soya bean architecture to obtain increased yields. Previous studies have shown that the expression levels of miR156s and their target genes are correlated with agronomic traits in crops, such as rice, switchgrass and alfalfa (Aung *et al*., 2014; Fu *et al*., 2012; Wang *et al*., 2015a). In this study, we also found a positive correlation between *GmmiR156b* expression level and ISA. The transgenic line *miR156b*OE‐5 with higher level of miR156b expression showed bigger statue, more branches and higher yield (Figure 1). Therefore, genetic control of miR156 expression levels is crucial for achieving ISA and high yield in soya bean.

*MiR156*s exert their functions by negatively regulating *SPL* genes. In rice and wheat, *IPA1* (*OsSPL14*) and its wheat ortholog *TaSPL17* are targets of *miR156* in the regulation of tillering and plant architecture (Jiao *et al*., 2010; Luo *et al*., 2012; Miura *et al*., 2010; Wang and Zhang, 2017). In Arabidopsis, *miR156* regulates branching through *SPL9* and *SPL15* (Schwarz *et al*., 2008). In soya bean, we obtained expression and preliminary genetic evidence in Arabidopsis showing that *GmSPL9d* may be the main target of *GmmiR156b* in branching (Figures 5b,c, 7c–e, and 6c). Because of the uniqueness of soya bean growth and development, further analysis of GmSPL9d loss of function mutations will confirm its major role in ISA of soya bean. Because several other *GmSPL* genes were also down‐regulated in the axillary buds of *GmmiR156b*OE soya bean plants (Figure 5c), we cannot exclude the possibility that *GmmiR156b* accomplishes its role in shoot architecture through several *SPL* genes. In plants, the *SPL* genes exist as a large gene family. In soya bean, we found that these *GmSPL* genes showed organ‐specific expression patterns and were differentially regulated by miR156b (Figure S6). It is conceivable that *GmmiR156s* control complex traits through a regulatory network composed of GmSPL and their downstream genes. Therefore, the *GmmiR156‐GmSPL* module is a key regulatory hub essential for soya bean ISA and high yield.

Symbiotic nitrogen fixation is a unique trait in soya bean, and number of nitrogen‐fixing nodule is one of the most important factors that determine the nitrogen fixation efficiency which plays an important role in grain yield and seed quality. It was previously reported that overexpression of *GmmiR156* using *rhizogenesis*‐mediated hairy root transformation significantly reduced nodule number in soya bean in a controlled growth condition (Yan *et al*., 2013). In this study, we found that several *GmSPL* genes including GmSPL9d were also highly expressed in mature nodules in soya bean (Figure S6), supporting the notion that the GmmiR156‐GmSPL module may also modulate nodulation and symbiotic nitrogen fixation of soya bean. It will be interesting to further investigate whether the overexpression of GmmiR156b affects nodulation and symbiotic nitrogen fixation in stable transgenic plants under controlled and natural environments, and how miR156b coordinately regulates symbiotic nitrogen fixation and ISA. It is also important to identify which target gene(s) are required for regulation of symbiotic nitrogen fixation.

Branching is suppressed by apical dormancy in most plants. Therefore, an optimal balance between apical growth and branching is critical to achieve an IPA and high yields. In rice and other plants, overexpression of *GmmiR156* results in increased tillers but stunted plants (Liu *et al*., 2017; Wang *et al*., 2015a). By contrast, *GmmiR156b* overexpression did not affect the activity of the soya bean apical meristem and primary shoot growth (Figures 1a,d and 3a). This finding implies that *GmmiR156b* does not mediate the activity of the apical meristem in soya bean; moreover, it is possible that the regulation of apical meristems and AMs is not tightly linked in soya bean. Genetic control of apical dominance is a fundamental question in plant biology, and extensive progress has been made using model plants such as Arabidopsis in past decades. Because of unique architecture of *miR156b*OE plants, it will be very interesting to dissect how *GmmiR156* regulate SAM and AM and uncover the molecular mechanisms underlying the ideal architectures of soya bean plants.

In this study, we produced several lines of evidence showing that *GmmiR156b* plays additional novel roles that contribute to the ideal architecture and high yields in soya bean. Firstly, overexpression of *GmmiR156b* resulted in plants with a sturdier stem (Figure 1e,f), which may protect plants against lodging. Secondly, *GmmiR156b*OE plants produced significantly more and bigger seeds (Figure 2c–e), resulting in substantially increased yields per plant (Figure 2f,g). Thirdly, *GmmiR156b* overexpression shortened the plastochron to increase trifoliolate leaves (Figure 3a–d), which might enhance photosynthetic activity to secure extra consumption by additional vegetative and reproductive organs. Finally, *GmmiR156b* did not affect the quality of soya bean seeds (Figure 2h,i). To our knowledge, we have identified novel roles for *GmmiR156b* in coordinating branching and plant apical growth that contribute to an ISA and regulate stem thickness, which in turn affects lodging resistance. This is also the first work to show that an ISA and high yields can be achieved by manipulating *GmmiR156b*. Our findings indicate a potential avenue for breeding high‐yielding soya bean plants with an ideal architecture. Our data also indicate the existence of a novel and conserved molecular mechanism by which SPLs complex with WUSs to determine plant architecture. Finally, our findings provide novel insight into the molecular mechanisms underlying species‐specific shoot architecture and high yield‐related traits, and they provide a strategy for the deliberate breeding of crops with an IPA and high yields.

## Experimental procedures

### Soya bean materials and growth conditions

Soya bean cultivar Williams 82 and two T_4_ transgenic *35S:miR156b* lines, *miR156b*OE‐5 and *miR156b*OE‐11 (Cao *et al*., 2015), were planted at the Experimental Station of Hebei Academy of Agriculture and Forestry Science (37°56′N, 114°43′E) in the summer of 2016 and 2017. Seeds were sown in fields with a proper soil moisture content (15%–20%) in three‐row plots with a row length of 6 m, adopting a completely random block design with three biological replicates. After 3 weeks, the seedlings were manually thinned to achieve interrow and interplant distances of 40, 50 or 60 cm, respectively. Plant architecture and yield‐related traits were evaluated during growth and at the harvest stage. Transgenic and wild‐type plants were grown in pots in a growth room under standard conditions (16‐h days, daytime temperature of 26 °C and night‐time temperature of 23 °C). Samples were collected at 15 DAE and used for gene expression analyses. Arabidopsis ecotype Col‐0 and the *spl9‐2* mutant (SALK_006573.53.25.x) used in this study were obtained from the Arabidopsis Resource Center (ABRC, Columbus, OH). Arabidopsis plants were grown under standard conditions (16‐h days, daytime temperature of 22 °C and night‐time temperature of 18 °C).

### Protein and lipid content determination

We used a MATRIX‐I FT‐NIR Spectrometer (Bruker Corp., Billerica, MA) to detect the protein and lipid contents in our plants after harvest. We used seeds from five plants of each *miR156b*OE line and wild type. Each plant was assessed three times.

### Plasmid construction for the generation of transgenic Arabidopsis plants

For *GmSPL9d* overexpression, pTF101 (a plant binary vector) harbouring *35S:GmSPL9d‐GFP* or *35S:7mGmSPL9d‐GFP* was generated. Full‐length (1080 bp) *GmSPL9d* cDNA was amplified and inserted into pTF101 using *Sma*I and *Bam*HI. For site‐directed mutagenesis, seven synonymous mutations in the *miR156b*‐binding site of *GmSPL9d* were designed as described by Jiao *et al*. (2010) and inserted into pTF101 using *Sma*I and *Bam*HI. Each construct was transformed into *Agrobacterium tumefaciens* strain GV3101 for Arabidopsis transformation using the floral dip method (Zhang *et al*., 2006). As pTF101 contains the *bar* gene, the transformants were screened on Murashige and Skoog medium containing Basta. The seeds were stratified in darkness at 4 °C for 2 days and then transferred to a culture room at 22 °C under a 16 h of light/8 h of dark photoperiod. At 7 days after stratification, the seedlings were transplanted to soil for molecular characterization and homozygote identification. Homozygous transgenic lines were used for phenotypic analyses. The primers used are summarized in Table S2.

### Y2H assays

Yeast transformation was performed following the manufacturer's instructions (Clontech Laboratories, Mountain View, CA). The full‐length coding sequences of *GmSPL9d* and *GmWUSa/b* and various deletion derivatives of *GmWUSa* were amplified with the listed primers (Table S2). The resulting products were cloned into pDONOR207 by the BP reaction (Invitrogen, Carlsbad, CA), and then into pGBKT7/pGADT7 by LR recombination (Invitrogen). The constructs were then cotransformed into *Saccharomyces cerevisiae* strain AH109. Yeast transformants were confirmed by growth on SD/‐Leu/‐Trp medium. The transformed yeast cells were incubated in liquid SD/‐Leu/‐Trp medium and grown at 28 °C to an optical density (OD_600_) value of 1, and the yeast cells were then collected and diluted to different ODs (0.1, 0.01, and 0.001). Protein–protein interactions were assayed in growth experiments using the suspended, transformed yeast on plates containing SD/‐Ade/‐His/‐Leu/‐Trp medium at 2–3 days after incubation at 28 °C.

### BiFC

The full‐length coding sequences of *GmSPL9d* and *GmWUSa/b* and all of the deleted derivatives of *GmWUSa* were cloned into the N‐terminus of yellow fluorescent protein (YFP) and the C‐terminus of YFP via Gateway reactions with the pDONOR vector system (Invitrogen). The primers used are listed in Table S2. The constructs were transformed in GV3101 cells, and the transformed clones were cultured and infiltrated into *N. benthamiana* leaves as described previously (Song *et al*., 2011). The infiltrated tobacco leaves were stained with 4′,6‐diamidino‐2‐phenylindole (DAPI) to detect the nucleus of each plant cell, and fluorescence was imaged using a Leica confocal laser scanning microscope (Leica Microsystems, Wetzlar, Germany) at 2 days after transformation. A 512 nm laser line was used to stimulate YFP, and an A530–585 bandpass filter (Leica Microsystems, Wetzlar, Germany) was used to collect the YFP signal.

### Protein expression constructs and protein purification

To purify recombinant GST‐GmSPL9d, MBP‐GmWUSa and MBP‐GmWUSb, the open‐reading frames of *GmSPL9d*, MBP‐*GmWUSa* and MBP‐*GmWUSb* were cloned into pGEX‐4T‐1 and pMAL‐c2x. The GST‐GmSPL9d and MBP‐GmWUSa expression plasmids were then transformed into *Escherichia coli* strain BL21. Protein purification was performed using glutathione agarose (Thermo Fisher Scientific, Waltham, MA) and amylose resin (NEB, Ipswich, MA) according to the manufacturers' instructions. The primers used to construct the plasmids are listed in Table S2.

### *In vitro* pull‐down assay

Purified GST and GST‐GmSPL9d were incubated with the same volume of GST beads in GST binding buffer for 2 h at room temperature, washed with GST binding buffer four times to remove redundant proteins and then incubated with MBP‐GmWUSa and MBP‐GmWUSb for another 2 h at room temperature. The cultures were then washed four times to remove redundant MBP‐GmWUSa or MBP‐GmWUSb. Samples were then collected, mixed with 2× SDS protein loading buffer and boiled for 10 min for Western blotting. Anti‐MBP (SAB2104172; Sigma‐Aldrich, St. Louis, MO) and anti‐GST (HT601‐01; Beijing TransGen Biotech Co., Ltd., Beijing, China) antibodies were used to detect GST‐ and MBP‐tagged proteins.

### Co‐IP

GmWUSa and GmWUSb in pDONOR207 were recombined with pEarlygate100 (35S:Flag‐pEarlygate) to generate the GmWUSa‐Flag fusion protein. *GmSPL9d* cDNA was recombined with pTF101 (with a GFP tag) to generate a *GmSPL9d‐GFP* fusion expression cassette. The constructs were introduced into *N. benthamiana* leaves through GV3101 infiltration. The leaves were harvested at 2 days after infiltration and frozen in liquid nitrogen. The frozen leaves were then homogenized and mixed with protein extraction buffer [50 mm Na_2_HPO_4_/NaH_2_PO_4_ (pH 7.4), 150 mm NaCl, 1% Triton X‐100, 15% glycerol, 1 mm phenylmethylsulfonyl fluoride (PMSF), and protease inhibitor cocktail (Roche, Basel, Switzerland)]. After protein extraction, flag beads (ANTI‐FLAG M2 Affinity Gel; Sigma‐Aldrich) were washed four times with phosphate‐buffered saline and incubated with the extracted proteins at room temperature for 1 h. The precipitated samples were washed at least four times with protein wash buffer [50 mm Na_2_HPO_4_/NaH_2_PO_4_ (pH 7.4), 150 mm NaCl, 0.1% Triton X‐100, 10% glycerol, 1 mm PMSF and protease inhibitor cocktail (Roche)] and then eluted with 2× SDS protein loading buffer and boiled for 10 min for Western blotting. The Flag‐ and GFP‐tagged proteins were detected with anti‐Flag antibodies [monoclonal ANTI‐FLAG M2‐Peroxidase (HRP) antibody; Sigma‐Aldrich] and anti‐GFP antibodies [Goat Polyclonal to GFP (HRP); Abcam, Cambridge, UK].

### Prediction of *GmmiR156b* targets and 5′ RACE mapping of miRNA cleavage sites

Putative targets of *miR156b* were predicted using psRNATarget (Dai and Zhao, 2011). For the 5′ RACE mapping of *miR156b* cleavage sites, total RNAs were isolated from a mixture of different organs collected from 4‐week‐old Williams 82 plants using Plant RNA Reagent (Invitrogen) according to the manufacturer's recommendations. A 5′‐Full RACE Kit with TAP (Takara Bio Inc., Kusatsu, Japan) was used to process the total RNAs and to map the 5′ termini of the primary transcripts. The cDNA samples were amplified by nested PCR according to the manufacturer's protocols. Gene‐specific primers (Table S2) were designed by Invitrogen.

### RNA extraction and quantitative PCR analysis

Total RNA and small RNAs were extracted using TRIzol reagent [Tiangen Biotech (Beijing) Co., Ltd., Beijing, China] and then treated with DNase I (Takara Bio Inc.) to remove contaminating genomic DNA. First‐strand cDNA was synthesized from the total RNA using a FastQuant RT Kit [Tiangen Biotech (Beijing) Co., Ltd.]. Stem‐loop‐specific reverse transcription for *miR156b* and *miR1520d* was performed as described previously (Chen *et al*., 2005). qRT‐PCR was performed using SuperReal PreMix Plus [SYBR Green; Tiangen Biotech (Beijing) Co., Ltd.]. The miRNA and specific primers for the genes analysed are listed in Table S2.

### Histology

The shoot apices of wild‐type plants at 15 DAE were fixed in 4% paraformaldehyde, dehydrated and embedded in Paraplast (Leica Microsystems). The wax‐embedded samples were then sectioned at a thickness of 8 μm. After being deparaffinized and dehydrated in a gradient ethanol series, the samples were stained with toluidine blue and scanned by microscopy.

### Scanning electron microscopy

For scanning electron microscopy, tissues were placed in 1% glutaraldehyde in 0.025 m phosphate buffer (sodium phosphate, pH 7.2–7.4) and a vacuum was applied for 10 min. The tissues were then fixed overnight at 4 °C. Next, the tissues were rinsed twice with 0.025 m phosphate buffer for 15 min, postfixed with 1% osmium tetroxide in 0.025 m phosphate buffer for 1 h at 4 °C and moved through a gradient ethanol series (20% increments), with each increment lasting 10 min and ending with two exchanges of 100% ethanol. Each tissue was then mounted to a test stub with double‐sided adhesive tape and sputter‐coated with a gold‐palladium alloy using an Eiko IB‐3 Ion Coater (Eiko Co., Ltd., Wakayama, Japan). The samples were then examined with a Hitachi S‐3500N Scanning Electron Microscope (Tokyo, Japan).

### *In situ* hybridization

A 380‐nt fragment of the *GmSPL9d* 5′‐untranslated region (UTR), a 153‐nt fragment of the *GmWUSa* 5′‐UTR and a 753‐nt fragment of the *GmHistone H4* coding sequencing were amplified from Williams 82 cDNA using KOD Polymerase (Stratagene, San Diego, CA) and cloned into pSPT 18 (Roche) using specific primers (Table S2). Digoxigenin‐labelled sense or antisense probes were synthesized with T7 or SP6 RNA Polymerase (Roche). Shoot apical tissues from wild‐type and transgenic materials at 15 DAE were dissected and fixed in 4% paraformaldehyde. Paraffin‐embedded materials were sectioned at a thickness of 8 μm. After the sections had been deparaffinized and dehydrated, hybridization and detection were performed as described previously (Long *et al*., 1996).

### Phylogenetic analysis

Seventeen homologous protein sequences of GmSPLs and Arabidopsis SPLs were obtained from Phytozome (www.phytozome.net) and TAIR (www.Arabidopsis.org) and imported into MEGA5 for complete alignment using Alignment Explorer/CLUSTAL (Tamura *et al*., 2011). A phylogenetic tree was then built with MEGA5 using the neighbour‐joining method with the bootstrapping value set at 1000 replications.

### Statistical analysis

All data were analysed using IBM SPSS Statistics 19 (IBM Corp., Armonk, NY). The means and standard errors of all results were calculated, and Student's *t*‐tests were performed to generate *P‐*values. SigmaPlot 10.0 (Systat Software Inc., San Jose, CA) was used to produce graphs.

## Conflict of interest

The authors declare that there is no conflict of interest regarding the publication of this article.

## Author contributions

ZS, YT and XL conceived the study and designed the experiments. ZS, CS, JY, QJ, LW and YW performed the experiments. FZ, BL and FK generated the transgenic soya bean materials. ZS, QZ, BZ, MZ and LZ conducted the field experiment. ZS, YW and LW analysed the data. XL wrote the manuscript with input from all of the authors.

## Supporting information


**Figure S1** *GmmiR156b* was overexpressed in different tissues of *miR156b*OE lines.
**Figure S2** Number of different type branches between WT and *miR156b*OE lines.
**Figure S3** Number of branches per plant at different planting densities.
**Figure S4** *GmmiR156b* overexpression decreased the length of the plastochron.
**Figure S5** Phylogenetic analysis of GmSPL proteins.
**Figure S6** Expression analysis of *GmSPLs* in different organs.
**Figure S7** Alignment between soybean GmSPL9d and Arabidopsis SPL.
**Figure S8** Interaction between GmSPL9d and various deleted derivatives of GmWUSa.
**Figure S9** WUS interacts with SPL9 in Arabidopsis.
**Figure S10** WUS interacts with SPL2 in Arabidopsis.

**Table S1** Target gene prediction of miR156b in psRNATarget.

**Table S2** Primers in this study.
