# Supplementary material for: Genetic improvement of the shoot architecture and yield in soya bean plants via the manipulation of GmmiR156b
Source: Plant Biotechnol J. 2018 May 23;17(1):50–62. doi: 10.1111/pbi.12946 (PMC6330639; doi:10.1111/pbi.12946)
Supplement: Supplementary file 1 — Figure S1 GmmiR156b was overexpressed in different tissues of miR156bOE lines. Figure S2 Number of different type branches between WT and miR156bOE lines. Figure S3 Number of branches per plant at different planting densities. Figure S4 GmmiR156b overexpression decreased the length of the plastochron. Figure S5 Phylogenetic analysis of GmSPL proteins. Figure S6 Expression analysis of GmSPLs in different organs. Figure S7 Alignment between soybean GmSPL9d and Arabidopsis SPL. Figure S8 Interaction between GmSPL9d and various deleted derivatives of GmWUSa. Figure S9 WUS interacts with SPL9 in Arabidopsis. Figure S10 WUS interacts with SPL2 in Arabidopsis. [file PBI-17-50-s001.docx]

**Supplementals**

**Figure S1**





**Figure S1.** *GmmiR156b* was overexpressed in different tissues of *miR156b*OE lines. Two independent transgenic plants of *miR156b*OE and wild-type plants were grown in a growth room and the relative levels of *miR156b* expression in the stem apex (SA), axillary bud (AB), stem (ST), leaves (L), roots (R), and nodes (N) were analyzed using qRT-PCR. The expression levels were normalized against the geometric mean of soybean *miR1520d*.

**Figure S2**

**
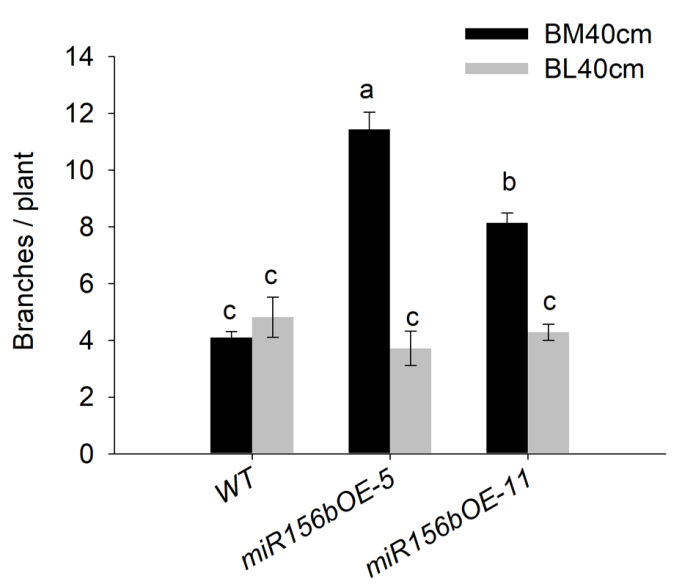
**

**Figure S2.** Number of different type branches between WT and *miR156b*OE lines. We divided the branching to two types according to the length of branch when we collected branching number. One type is BM40cm which means the length of branch more than 40cm. Another type is BL40cm which means the length of branch less than 40cm. The data in the graphs represent means ± SEs, (n=20). Different letters indicate a significant difference (Student-Newman-Kuels test; *P* < 0.005).

**Figure S3**


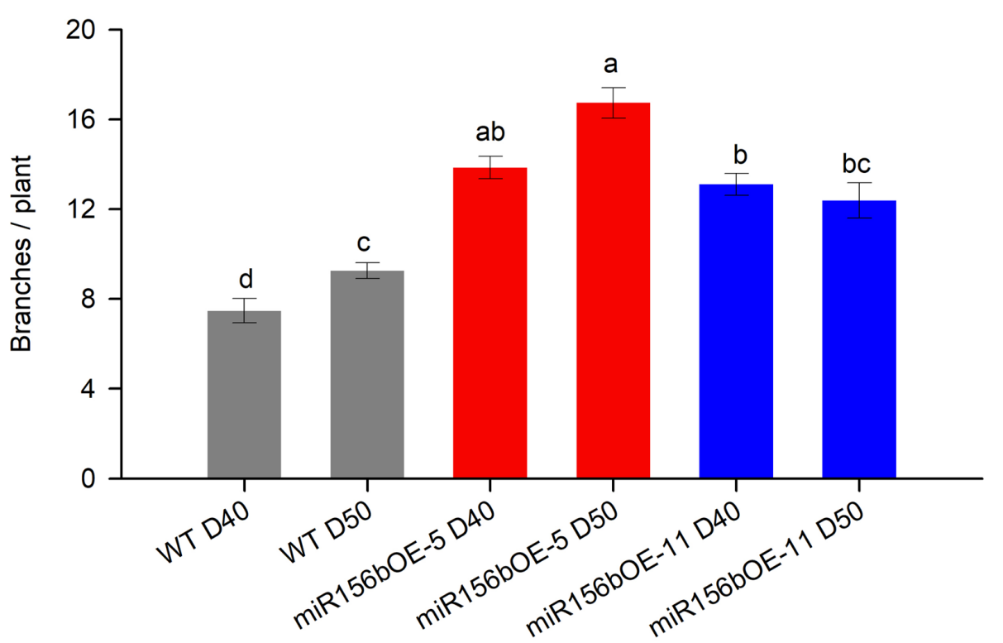


**Figure S3.** Number of branches per plant at different planting densities. The different genotypes were planted at three plant densities as a function of in-row plant spacing (40, 50, and 60 cm) in 6-m long rows in a plot. The different densities and means of the total branches per *miR156b*OE and wild-type (WT) plant were evaluated (n > 10). The data in the graphs represent means ± SEs. Different letters indicate a significant difference (Student-Newman-Kuels test; *P* < 0.005). ‘D40’ and ‘D50’ represent planting densities with row spacing of 40 and 50 cm, respectively.

**Figure S4**


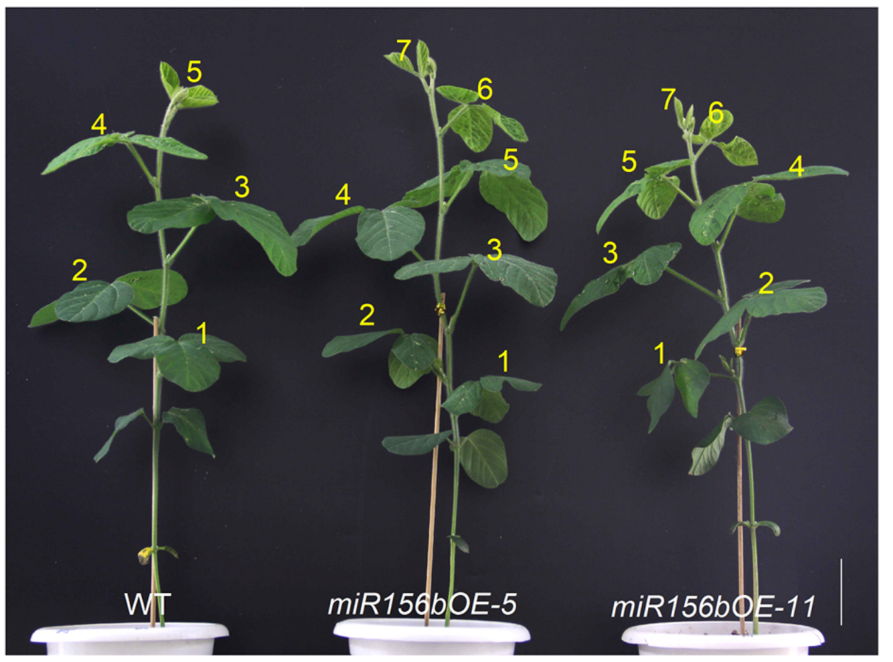


**Figure S4.** *GmmiR156b* overexpression decreased the length of the plastochron. A phenotypic analysis of trifoliate leaves from *miR156b*OE-5, *miR156b*OE-11, and wild-type plants grown in a growth room at 30 DAE from soil was conducted. Numbers show the order of trifoliolate leaves formed. Scale bar, 5 cm.

**Figure S5**


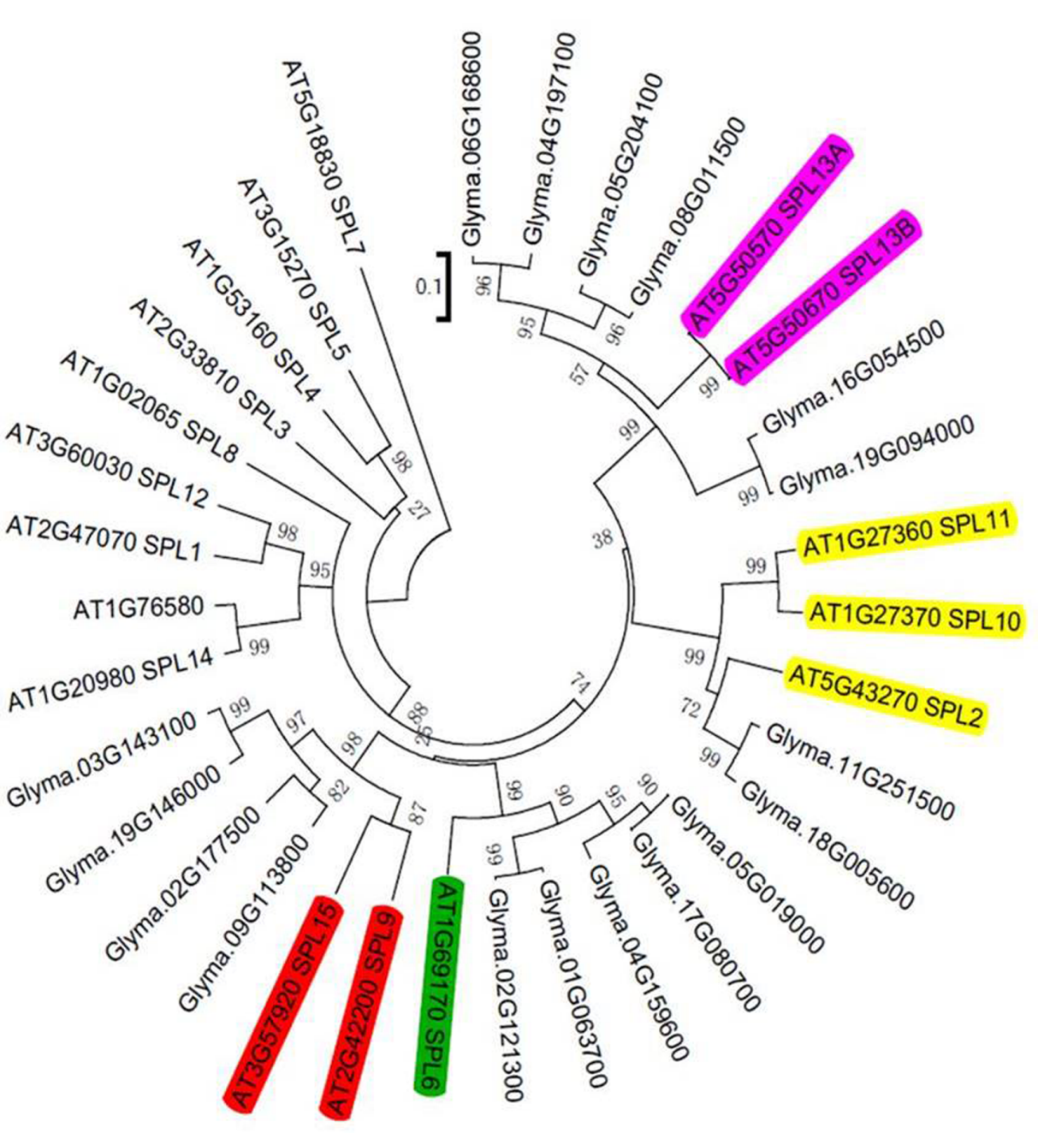


**Figure S5.** Phylogenetic analysis of GmSPL proteins. A MEGA5-based neighbor-joining tree was inferred from the amino acid sequences of 17 predicted target genes of *miR156b* in soybean and SPL family proteins in Arabidopsis. Different colors represent the different homologs of SPL family proteins.

**Figure S6**

**
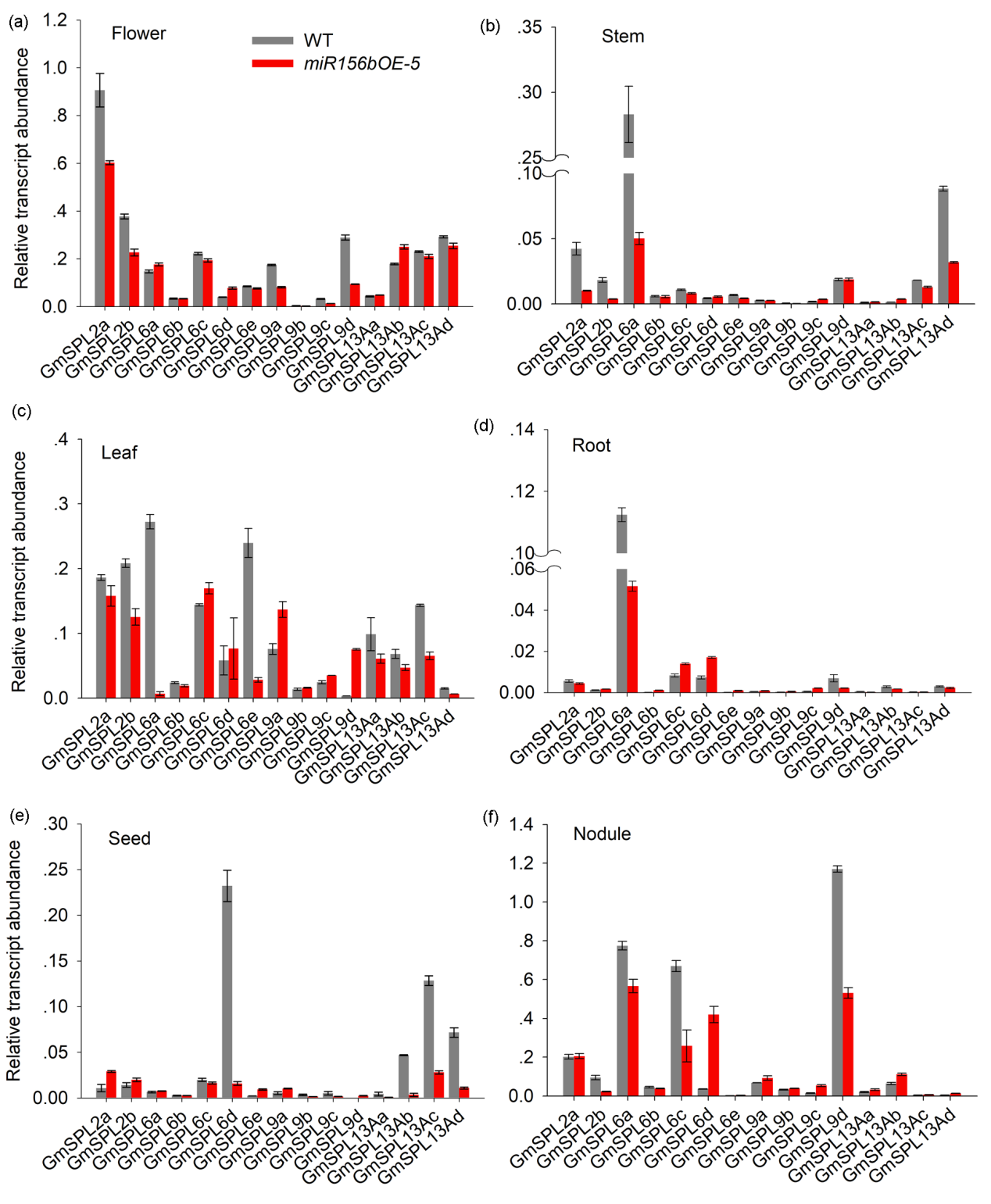
**

**Figure S6.** Expression analysis of *GmSPLs* in different organs. qRT-PCR analysis of *GmSPLs* expression in flower (a), stem (b), leaf (c), root (d), seed (e) and nodule (f) of miR156bOE-5 and wild-type plants. The *GmELF1b* gene was used as an endogenous control for gene expression.

**Figure S7**


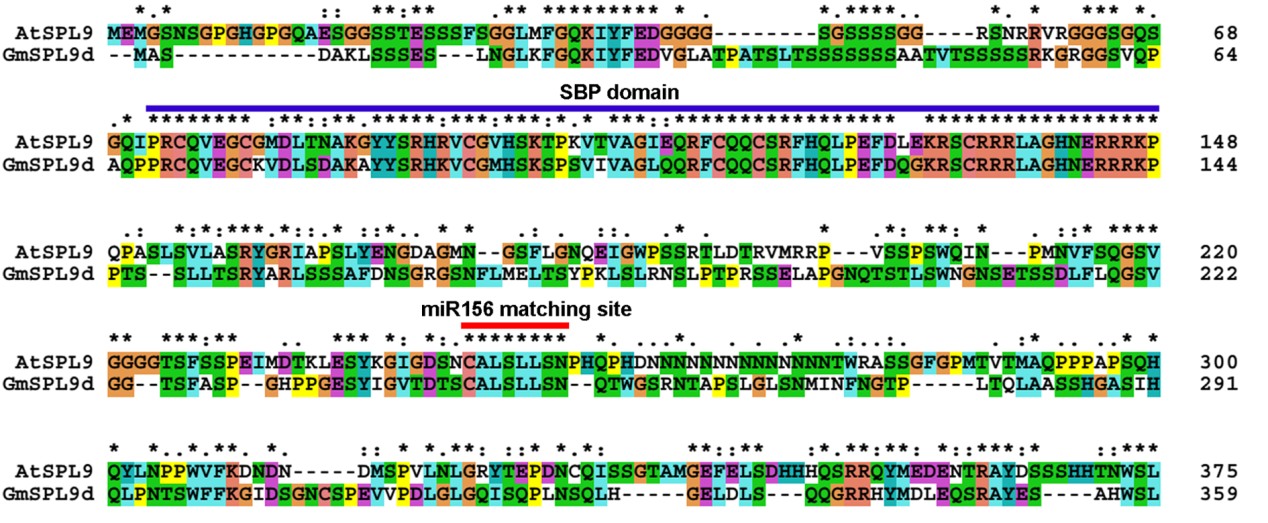


**Figure S7.** Alignment between soybean GmSPL9d and Arabidopsis SPL. The peptide sequences of GmSPL9d and Arabidopsis SPL were aligned using Clustal X. Stars indicate amino acids conserved in the two SPL proteins. The region covered by the blue line is a conserved SPL domain. The amino acids covered by a red line are the *miR156* matching site.

**Figure S8**

**
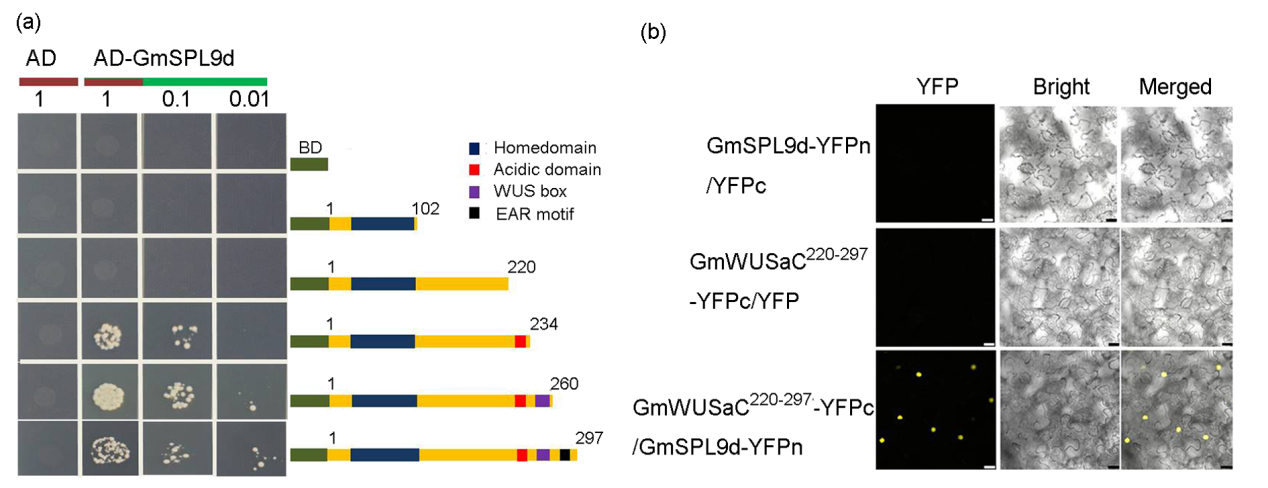
**

**Figure S8.** Interaction between GmSPL9d and various deleted derivatives of GmWUSa. (a) Y2H assay validating the interaction of GmSPL9d with various deleted derivatives of GmWUSa. Right, box diagrams of the GmWUSa derivatives. Colored boxes indicate different functional domains. Numbers indicate the amino acid residues. (b) BiFC assay in tobacco leaves showing the interaction between GmSPL9d and GmWUSaC^220–297^. Panels (left to right): YFP; bright; merged channels. Scale bar, 25 μm.

**Figure S9**


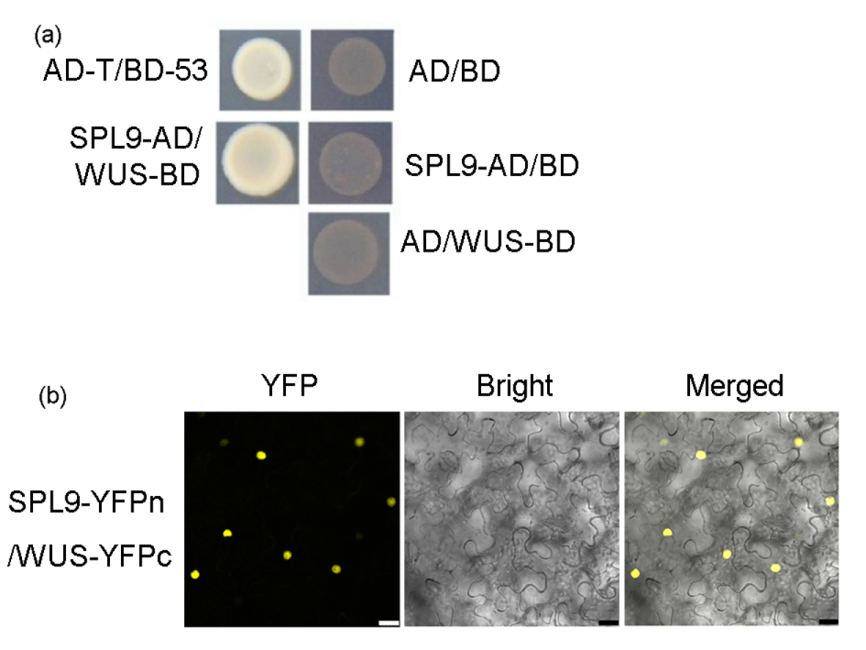


**Figure S9.** WUS interacts with SPL9 in Arabidopsis. (a) Y2H assay results validating the interaction of SPL9 with WUS. SPL9 and WUS were fused to the AD and BD, respectively. AD-T interacted with BD-53 as a positive control. The AD and BD were used as negative controls. (b) Results of a BiFC assay validating the interaction of SPL9 with WUS. SPL9 was fused with YFP^N^ (N-terminus of YFP) and WUS was fused with YFP^C^ (C-terminus of YFP). Visible light indicates the interaction between SPL9 and WUS in the nucleus. Panels (left to right): YFP; bright; merged channels. Scale bar, 25 μm.

**Figure S10**


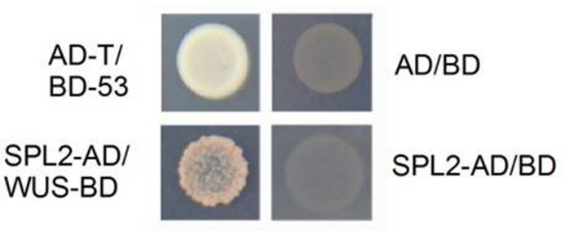


**Figure S10.** WUS interacts with SPL2 in Arabidopsis. (a) Y2H assay results validating the interaction of SPL9 with WUS. SPL2 and WUS were fused to the AD and BD, respectively. AD-T interacted with BD-53 as a positive control. The AD and BD were used as negative controls.
