# Supplementary material for: Genetic improvement of the shoot architecture and yield in soya bean plants via the manipulation of GmmiR156b
Source: Plant Biotechnol J. 2018 May 23;17(1):50–62. doi: 10.1111/pbi.12946 (PMC6330639; doi:10.1111/pbi.12946)
Supplement: Supplementary file 2 — Table S1 Target gene prediction of miR156b in psRNATarget. [file PBI-17-50-s002.docx]

| **Table S1 Target gene prediction of miR156b in psRNATarget.** | | | | | | |
| --- | --- | --- | --- | --- | --- | --- |
| **Gene** | **Alias** | **Domain** | **Expectation** | **Multiplicity** | **Inhibition** | **Cleavage site** |
| *Glyma.11G251500* | *GmSPL2a* | SBP | 0 | 1 | Cleavage | CDS |
| *Glyma.18G005600* | *GmSPL2b* | SBP | 0 | 1 | Cleavage | CDS |
| *Glyma.02G121300* | *GmSPL6a* | SBP | 0 | 1 | Cleavage | CDS |
| *Glyma.01G063700* | *GmSPL6b* | SBP | 0 | 1 | Cleavage | CDS |
| *Glyma.05G019000* | *GmSPL6c* | SBP | 0 | 1 | Cleavage | CDS |
| *Glyma.17G080700* | *GmSPL6d* | SBP | 0 | 1 | Cleavage | CDS |
| *Glyma.04G159600* | *GmSPL6e* | SBP | 0 | 1 | Cleavage | CDS |
| *Glyma.02G177500* | *GmSPL9a* | SBP | 0 | 1 | Cleavage | CDS |
| *Glyma.09G113800* | *GmSPL9b* | SBP | 0 | 1 | Cleavage | CDS |
| *Glyma.03G143100* | *GmSPL9c* | SBP | 0 | 1 | Cleavage | CDS |
| *Glyma.19G146000* | *GmSPL9d* | SBP | 0 | 1 | Cleavage | CDS |
| *Glyma.05G204100* | *GmSPL13Aa* | SBP | 1 | 1 | Cleavage | CDS |
| *Glyma.08G011500* | *GmSPL13Ab* | SBP | 1 | 1 | Cleavage | CDS |
| *Glyma.06G168600* | *GmSPL13Ac* | SBP | 1 | 1 | Cleavage | CDS |
| *Glyma.04G197100* | *GmSPL13Ad* | SBP | 1 | 1 | Cleavage | CDS |
| *Glyma.16G054500* | *GmSPL13Ba* | SBP | 1 | 1 | Translation |  |
| *Glyma.19G094000* | *GmSPL13Bb* | SBP | 1 | 1 | Translation |  |
| Note:psRNATarget: a plant small RNA target analysis server. | | | |  |  |  |
| http://plantgrn.noble.org/psRNATarget/. Multiplicity indicates the the number of miRNA/target site pairs. | | | | | | |
|  |  |  |  |  |  |  |
