# Supplementary material for: Genetic improvement of the shoot architecture and yield in soya bean plants via the manipulation of GmmiR156b
Source: Plant Biotechnol J. 2018 May 23;17(1):50–62. doi: 10.1111/pbi.12946 (PMC6330639; doi:10.1111/pbi.12946)
Supplement: Supplementary file 3 — Table S2 Primers in this study. [file PBI-17-50-s003.docx]

| **Table S2 Primers in this study.** | | |
| --- | --- | --- |
| **Name** | **Sequence** | **Function** |
| 35S:miR156b-Forward | GCTCTAGAGGGTTCTATTGGTGGTTG | Construction for transformation |
| 35S:miR156b-Reverse | CGAGCTCCGTCTACTTTGGCTAGAAAG | Construction for transformation |
| 35S:(7m)SPL9d-GFP-Forward | TCCCCCCGGG ATGGCTTCAGACGCCAAAC | Construction for transformation, CoIP |
| 35S:(7m)SPL9d-GFP-Reverse | CGGGATCCTTAAAGTGACCAATGAGCAGATTC | Construction for transformation, CoIP |
| GST-GmSPL9d-Forward | GGATCCATGGCTTCAGACGCCAAAC | Construction for fused protein |
| GST-GmSPL9d-Reverse | GAATTCAAGTGACCAATGAGCAGATTC | Construction for fused protein |
| MBP-GmWUSa-Forward | CGGAATTC ATGATGGAACCTCAACAACAAC | Construction for fused protein |
| MBP-GmWUSa-Reverse | CGGGATCC AGCATAATCTGGAGACCTGC | Construction for fused protein |
| gateway-GmSPL9d-Forward | GGGGACAAGTTTGTACAAAAAAGCAGGCTTCATGGCTTCAGACGCCAAAC | Construction for Y2H, BiFC |
| gateway-GmSPL9d-Reverse | GGGGACCACTTTGTACAAGAAAGCTGGGTCAAGTGACCAATGAGCAGATTCAT | Construction for Y2H, BiFC |
| gateway-GmWUSa-Forward | GGGGACAAGT TTGTACAAAAAAGCAGGCTTC ATGATGGAACCTCAACAACAAC | Construction for Y2H, BiFC, CoIP |
| gateway-GmWUSa-Reverse | GGGGACCACTTTGTACAAGAAAGCTGGGTCAGCATAATCTGGAGACCTGC | Construction for Y2H, BiFC, CoIP |
| gateway-GmWUSb-Forward | GGGGACAAGT TTGTACAAAAAAGCAGGCTTC ATGATGGAACCTCAACAACAAC | Construction for Y2H, BiFC, CoIP |
| gateway-GmWUSb-Reverse | GGGGACCACTTTGTACAAGAAAGCTGGGTCAGCATAATCTGGAGACCTGC | Construction for Y2H, BiFC, CoIP |
| gateway-GmWUSaD102-Reverse | GGGGACCACTTTGTACAAGAAAGCTGGGTC AGTGAACCTTTTCTTCTGCCTT | Construction for Y2H |
| gateway-GmWUSaD220-Reverse | GGGGACCACTTTGTACAAGAAAGCTGGGTCTTGATCACTTGGCCTTATTTTG | Construction for Y2H |
| gateway-GmWUSaD234-Reverse | GGGGACCACTTTGTACAAGAAAGCTGGGTCCTTAGTAGCACCAATGTTCTCTGCT | Construction for Y2H |
| gateway-GmWUSaD260-Reverse | GGGGACCACTTTGTACAAGAAAGCTGGGTCGTTATACGAATTAGACTTGAGGTTG | Construction for Y2H |
| gateway-GmWUSaD221-Forward | GGGGACAAGTTTGTACAAAAAAGCAGGCTTCATGGAAACCCTTGAAGAAGAAG | Construction for Y2H, BiFC |
| gateway-SPL9-Forward | GGGGACAAGTTTGTACAAAAAAGCAGGCTTC ATGGAGATGGGTTCCAACTC | Construction for Y2H, BiFC |
| gateway-SPL9-Reverse | GGGGACCACTTTGTACAAGAAAGCTGGGTC GAGAGACCAGTTGGTATGGTGAG | Construction for Y2H, BiFC |
| gateway-WUS-Forward | GGGGACAAGTTTGTACAAAAAAGCAGGCTTC ATGGAGCCGCCACAGC | Construction for Y2H, BiFC |
| **Name** | **Sequence** | **Function** |
| gateway-WUS-Reverse | GGGGACCACTTTGTACAAGAAAGCTGGGTC GTTCAGACGTAGCTCAAGAGAAG | Construction for Y2H, BiFC |
| SL-gma-miR156b-RT | GTCGTATCCAGTGCAGGGTCCGAGGTATTCGCACTGGATACGAC TGTGCT | Reverse transcript |
| SL-gma-miR1520d-RT | GTCGTATCCAGTGCAGGGTCCGAGGTATTCGCACTGGATACGACTTGTCA | Reverse transcript |
| RACE-Glyma.11G251500-in | ATCAGTGTGCCAATACTCTGC | 5'RACE |
| RACE-Glyma.11G251500-out | AGGAAAAGGTGATTCATAGGG | 5'RACE |
| RACE-Glyma.18G005600-in | ATCAGTGTGCCAATACTCTGC | 5'RACE |
| RACE-Glyma.18G005600-out | AGGAAAAGGTGATTCATAGGG | 5'RACE |
| RACE-Glyma.02G121300-in | TCGGCTGTCTCTGTCGGGTAT | 5'RACE |
| RACE-Glyma.02G121300-out | AGATTGTATCACATGGAAAGGACTC | 5'RACE |
| RACE-Glyma.01G063700-in | ATTTGACCATCTCTGTTGGGTATGC | 5'RACE |
| RACE-Glyma.01G063700-out | ATCTATTCTTGATAACCTCCTTGG | 5'RACE |
| RACE-Glyma.05G019000-in | ATCTTTTCAGAGACCTGACTCATGT | 5'RACE |
| RACE-Glyma.05G019000-out | CATGATTTAGTTCTGATGGAAAACT | 5'RACE |
| RACE-Glyma.17G080700-in | TGAGCTTATTCCTATCTTGTCAGAG | 5'RACE |
| RACE-Glyma.17G080700-out | CTGGTGGAAAACTATCTGACACTCG | 5'RACE |
| RACE-Glyma.04G159600-in | TTGTAATGGCTGTGGCTGCTT | 5'RACE |
| RACE-Glyma.04G159600-out | GGAAACCTATCTGACACTCCACT | 5'RACE |
| RACE-Glyma.02G177500-in | GCAAAGACGCCACAGCAAAT | 5'RACE |
| RACE-Glyma.02G177500-out | CTCCCTTTAGGTACAACCATGAGGT | 5'RACE |
| RACE-Glyma.09G113800-in | TGTGTCATGGATGTCCCATTG | 5'RACE |
| RACE-Glyma.09G113800-out | AATCACCAGAACCAACTCCCTTTAG | 5'RACE |
| RACE-Glyma.03G143100-in | CACCATGAGATGATGCAGCAG | 5'RACE |
| RACE-Glyma.03G143100-out | ATCTGACCGAGACCTAGATCAGG | 5'RACE |
| RACE-Glyma.19G146000-in | GACCGAGACCTAGATCAGGGACCA | 5'RACE |
| **Name** | **Sequence** | **Function** |
| RACE-Glyma.19G146000-out | GTTCCACAAATGAGGCAAGGCTAC | 5'RACE |
| RACE-Glyma.05G204100-in | ATCATTGGGGCCCAAAAC | 5'RACE |
| RACE-Glyma.05G204100-out | CCATTGAAAGGGAAATAGTGG | 5'RACE |
| RACE-Glyma.08G011500-in | GAGGAATGGTCACTCCCG | 5'RACE |
| RACE-Glyma.08G011500-out | CCATTGAAAGGGAAATAGTGG | 5'RACE |
| RACE-Glyma.06G168600-in | GCCAAGCTATATATGGAGGAACAAT | 5'RACE |
| RACE-Glyma.06G168600-out | TGCAGTGGGATCCATGATGATCAG | 5'RACE |
| RACE-Glyma.04G197100-in | AGACTCTAAGCTGTTATTATCCTGC | 5'RACE |
| RACE-Glyma.04G197100-out | TCCATGATCAGAAGCCAAGCTATAC | 5'RACE |
| gma-Histone 4-in situ-Forward | ATCCAAATTCAAGTTCGCAC | in situ hybridization |
| gma-Histone 4-in situ-Reverse | TCCAGCTTACCCACCGTC | in situ hybridization |
| gma-WUSa-in situ-Forward | CCTATTATAAGTTTATAACCCCCCT | in situ hybridization |
| gma-WUSa-in situ-Reverse | CACTACTCAGAACAAACAAAATGGT | in situ hybridization |
| gma-SPL9d-in situ-Forward | AAGAAGAGAGGGTACAATCTGTCTC | in situ hybridization |
| gma-SPL9d-in situ-Reverse | GTGCTGGTGATAAGGAAAGGTAG | in situ hybridization |
| gma-miR156b-qPCR-Forward | CGGGCCTGACAGAAGAGAGAG | qRT-PCR |
| gma-miR1520d-qPCR-Forward | CGGACCATCAGAACATGACACG | qRT-PCR |
| gma-miRNA-qPCR-universal-Reverse | GTGCAGGGTCCGAGGT | qRT-PCR |
| gma-GmSPL2a-qPCR-Forward | CACACTTTAGGTTTACCAGGGCAAT | qRT-PCR |
| gma-GmSPL2a-qPCR-Reverse | AATCCAACAAATCAGTGGACTCCAC | qRT-PCR |
| gma-GmSPL2b-qPCR-Forward | GGCAAGATATGCACAGCAGCAAG | qRT-PCR |
| gma-GmSPL2b-qPCR-Reverse | CCATTTGGAACTTCCATACCTGGA | qRT-PCR |
| gma-GmSPL6a-qPCR-Forward | TCAGAGGGTGGAGCAGCAAAG | qRT-PCR |
| gma-GmSPL6a-qPCR-Reverse | CCCACTAGGCCACTATTATAGCATC | qRT-PCR |
| **Name** | **Sequence** | **Function** |
| gma-GmSPL6b-qPCR-Forward | TTCTCTAGTTGAGTCCAAGACTCAGG | qRT-PCR |
| gma-GmSPL6b-qPCR-Reverse | CGAAAGCGTGGTCGGATGTA | qRT-PCR |
| gma-GmSPL6c-qPCR-Forward | TGACAGACACGCCTACGCCTAT | qRT-PCR |
| gma-GmSPL6c-qPCR-Reverse | TCTGCAGACAAGTTCTTGAACCG | qRT-PCR |
| gma-GmSPL6d-qPCR-Forward | GTCTTGCACCCCGACCTGG | qRT-PCR |
| gma-GmSPL6d-qPCR-Reverse | AGAGTGTTGGGAGGTGAGGGTG | qRT-PCR |
| gma-GmSPL6e-qPCR-Forward | TCTTCTTCCGAGTTGTCGACG | qRT-PCR |
| gma-GmSPL6e-qPCR-Reverse | AGTCTTGGAGTGAACCTCACAAACT | qRT-PCR |
| gma-GmSPL9a-qPCR-Forward | CCAGGGCCTGTGAGTCTTCTC | qRT-PCR |
| gma-GmSPL9a-qPCR-Reverse | TAGGCACAGATTCCCCTCATACT | qRT-PCR |
| gma-GmSPL9b-qPCR-Forward | TTGATCAGTTACCAGACTGGCACT | qRT-PCR |
| gma-GmSPL9b-qPCR-Reverse | CCTTGCTGATATGTTCAAACAAACC | qRT-PCR |
| gma-GmSPL9c-qPCR-Forward | CACCCGACTGTGTTTTGTTAGTGTC | qRT-PCR |
| gma-GmSPL9c-qPCR-Reverse | TCTTAGGCGAGCAATGTGTGC | qRT-PCR |
| gma-GmSPL9d-qPCR-Forward | ACACAGGTATGGTGTTTTCTGCC | qRT-PCR |
| gma-GmSPL9d-qPCR-Reverse | TAGCAAACATCAAAGAGGCTGTC | qRT-PCR |
| gma-GmSPL13Aa-qPCR-Forward | TCCTTTATTTGACTGTCCAGTGGC | qRT-PCR |
| gma-GmSPL13Aa-qPCR-Reverse | TGAAAGAGGTCCAAGGAAACCG | qRT-PCR |
| gma-GmSPL13Ab-qPCR-Forward | CTCTCTTCTGTCATCACCACCACAG | qRT-PCR |
| gma-GmSPL13Ab-qPCR-Reverse | GATGCATTGATGAGGAATGGTCACT | qRT-PCR |
| gma-GmSPL13Ac-qPCR-Forward | GGTTAGCCCAGATTGGAGTGG | qRT-PCR |
| gma-GmSPL13Ac-qPCR-Reverse | CCTTCTTTGTGAGAAGATCCGAGA | qRT-PCR |
| gma-GmSPL13Ad-qPCR-Forward | CCCACAAAGAAGGGAAGCAAG | qRT-PCR |
| gma-GmSPL13Ad-qPCR-Reverse | CGAACGGAAGAGGATGGAAAAG | qRT-PCR |
| **Name** | **Sequence** | **Function** |
| gma-GmELF1b-qPCR-Forward | GTTGAAAAGCCAGGGGACA | qRT-PCR |
| gma-GmELF1b-qPCR-Reverse | TCTTACCCCTTGAGCGTGG | qRT-PCR |
| gma-GmSPL9d(CDS)-qPCR-Forward | TTCGTCTGCTTTTGATAATAGTGGC | Checking transgenic Arabidopsis |
| gma-GmSPL9d(CDS)-qPCR-Reverse | GATGATGTCTCTGAGTTGCCATTCC | Checking transgenic Arabidopsis |
| gma-Actin8-qPCR-Forward | TCAGCACTTTCCAGCAGATG | Checking transgenic Arabidopsis |
| gma-Actin8-qPCR-Reverse | ATGCCTGGACCTGCTTCAT | Checking transgenic Arabidopsis |
